# Insights into Chemical Bonding Modes and Heat Transport at the Molecular Level

**DOI:** 10.1021/acs.jpclett.4c02325

**Published:** 2024-10-31

**Authors:** Shintaro Fujii, Yoshiaki Shoji, Yuma Masuda, Takanori Fukushima, Tomoaki Nishino

**Affiliations:** † Department of Chemistry, School of Science, Tokyo Institute of Technology, 2-12-1 W4-10 Ookayama, Meguro-ku, Tokyo 152-8551, Japan; ‡ Laboratory for Chemistry and Life Science, Institute of Innovative Research, Tokyo Institute of Technology, 4259 Nagatsuta, Midori-ku, Yokohama 226-8501, Japan; # Research Center for Autonomous Systems Materialogy (ASMat), Institute of Innovative Research, Tokyo Institute of Technology, 4259 Nagatsuta, Midori-ku, Yokohama 226-8501, Japan

## Abstract

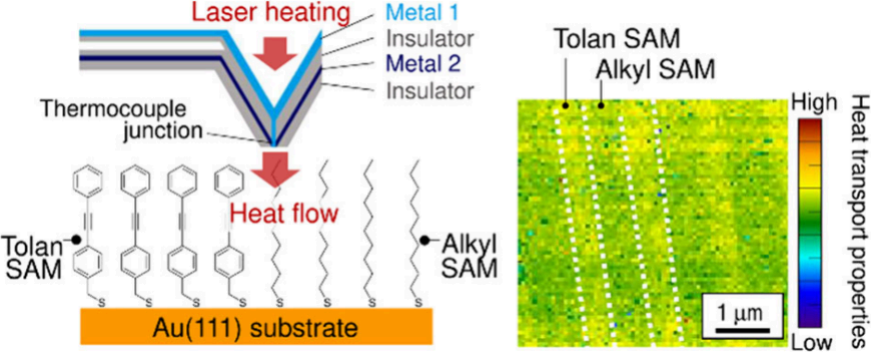

Despite the demand for nanoscale thermal management technologies of material surfaces and interfaces using organic molecules, heat transport properties at the single molecular level remain elusive due to the experimental difficulty of measuring temperature at the nanoscopic scale. Here we show how chemical bonding modes can affect the heat transport properties of single molecules. We focused on four molecular systems: benzylthiol linked to another phenyl group by either a triple (compound **1**), double (**3**), or amide (**4**) bond and a common linear alkanethiol (**2**), all of which are nearly identical in molecular length. We prepared binary self-assembled monolayers (SAMs) using **1** as a common reference in combination with **2**–**4** and investigated their relative heat transport properties using scanning thermal microscopy (SThM). Two-dimensional temperature mapping of the binary SAMs showed that C≡C and C=C bonds provide more effective pathways for heat transport compared to C–C bonds. Since the amide molecule has resonance structures with C=N double bond character, we expected that its heat transport properties would be comparable to those of the thiols containing triple or double bonds. However, the heat transport properties of this molecule prevailed over the others, most likely due to the formation of additional heat transport pathways caused by intermolecular hydrogen bonding. These findings may provide important guidelines for the design of organic materials for nanoscale thermal management.

Heat is a ubiquitous form of energy,^1^ but in a sense, it can also be thought of as the residue of electromagnetic and electronic energy that cannot be extracted as work. Heat is dissipative, making it difficult to use and control. In particular, heat transport in organic materials consisting of molecules remains elusive^2,3^ and unexplored, in sharp contrast to recent advances in understanding heat transport in metals and inorganic materials. One of the major obstacles is the lack of experimental knowledge needed to understand heat transport at the molecular level,^4−7^ which is the fundamental building block of organic materials. Conversely, if the behavior of heat transport through molecules can be elucidated, it will be possible to not only promote an essential understanding of heat transport in bulk organic and polymeric materials, which are assemblies of molecules, but also to develop technologies for nanoscale thermal management through surface or interface modifications using monolayers or thin films of organic molecules.

In the past, Gotsmann and co-workers have reported the effect of molecular species on heat transport at the single molecule level, focusing on the molecular backbone.^6,7^ Using an STM setup, heat transport properties of a dithiol-terminated oligo(phenylene ethynylene) derivative (OPE), sandwiched between a Au tip and a Au substrate, were measured and compared with those of *n*-octanedithiol (HS-(CH_2_)_*n*_-SH, *n* = 8).^6^ The single-molecule thermal conductivity of dithiol-terminated OPEs (*ca*. 20 pW K^–1^) was found to be smaller than that of *n*-octanedithiol (*ca*. 40 pW K^–1^). To the best of our knowledge, experimental studies focusing on molecular backbone and examined using local probe methods have been limited to these examples.

Here we report a systematic study directly comparing the heat transport properties of binary self-assembled monolayers (SAMs) fabricated using a combination of two types of molecules with different backbones, evaluated by scanning thermal microscopy (SThM).^8−15^ We clarified the differences in heat transport properties along the long axis of molecules in four molecular systems including simple alkyl, stilbene, tolan, and *N*-phenylbenzamide backbones (Figure 1).

**Figure 1:**
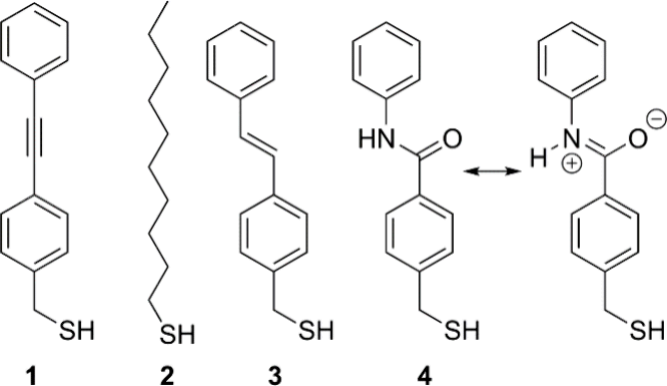
Chemical structures of the thiols used in the present study. For **4**, the resonance structures of the amide moiety are shown.

We have recently reported SThM imaging of temperature distributions for binary SAMs having two separate domains consisting of an alkanethiol (butanethiol or hexadecanethiol) and a benzenthiol.^8^ Although visualization of nanoscale temperature distributions by SThM has been applied to the surfaces of metals and inorganic materials, its utility in temperature imaging of organic monolayers has rarely been investigated. This previous study, which was the first to successfully visualize differences in the heat transport properties of SAM domains, demonstrated that SThM is a reliable method for evaluating the nanoscale thermal transport properties of organic molecules. In the present study, we conducted SThM imaging on patterned binary SAMs consisting of diphenyl ethyne derivative (**1**)/*n*-decanethiol (**2**), **1**/diphenyl ethene derivative (**3**), and **1**/phenyl benzamide derivative (**4**) on Au(111) (Figure 1). The constituent molecules have similar molecular lengths, while the chemical bonding modes are different, i.e., single, double, amide, and triple bonds.

The patterned binary SAMs on Au(111) were prepared by a μ-contact printing method^16^ according to the procedure reported previously.^8^ The μ-contact printing (μ-CP) method is a type of soft lithography, in which a surface pattern of polydimethylsiloxane (PDMS) transferred from a mold is modified with a molecular solution, and the soft contact between the PDMS and the substrate surface prints a pattern of SAM domains on the substrate surface. After printing, the substrate is immersed in another molecular solution, resulting in a surface pattern of binary SAM domains (Figure S1). Typically, AFM topographic and friction^17^ images of a patterned binary SAM with the domains of **1** and **2** (**1**/**2** SAM) display weak and distinct molecular contrast in terms of height and friction signals, respectively (Figure 2a,b). In the friction image of this sample, on which **1** and **2** are chemically adsorbed by Au–S bonding, linear domains with widths of ∼2 μm can be seen, and the deep holes and flat terraces of the underlying Au(111) are observed in the topographic image.^18,19^ The narrow region corresponds to the domain of **1** formed by the μ-CP process, while the wide linear region represents the domain of **2** formed after the solution process (Figure S1). According to the friction contrast, the domain of **1** has a greater frictional force than that of **2** (Figure 2b). This observation is consistent with a previous literature,^20^ which reports that the magnitude of the friction force is directly correlated with the size of the terminal groups, and a phenyl group-terminated SAM exhibits greater friction than a methyl group-terminated one.

**Figure 2:**
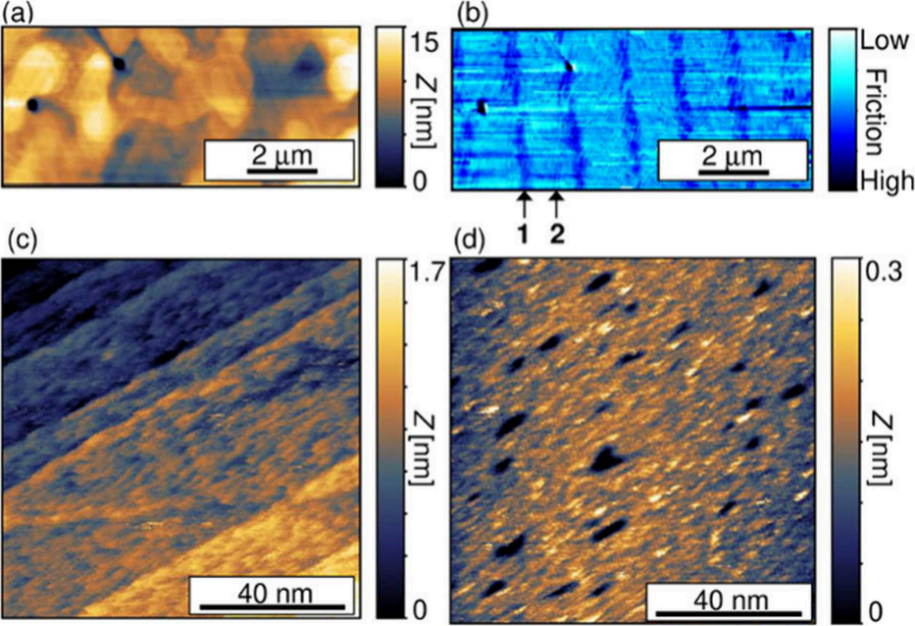
(a, b) AFM (a) topographic and (b) friction images of a patterned binary **1**/**2** SAM. (c, d) STM images of local areas corresponding to domains of (c) **1** and (d) **2** for the **1**/**2** SAM. The images were recorded in constant current mode using a sample bias voltage of +1.2 V and a tunneling current of 200 pA.

Along with the AFM observations, STM imaging of binary **1**/**2** SAM further confirmed the 2D pattern formation of two domains. Figure 2c,d shows STM images for magnified areas of the SAM domains. The nanosized bright spots correspond to individual molecules adsorbed on the Au(111) surface. The domain of **1** (Figure 2c) displays a less ordered structure without clear molecular contrast, which is typical of SAMs of tolan derivatives.^21^ In contrast, the bright spots in the domain of **2** are more distinct, with clearly visible small depressions (black holes) corresponding to one-Au-atom-deep etch pits in which molecules of **2** are adsorbing.^22,23^ Likewise, patterned binary SAMs of **1**/**3** and **1**/**4** were characterized by AFM imaging (Figure S2). The friction images of binary **1**/**3** and **1**/**4** SAMs did not show clear image contrasts, reflecting the fact that the terminal groups of **1**, **3**, and **4** are identical. The STM (Figure 2) and AFM (Figure S2) images show that the molecular density is comparable across the different binary SAM systems studied, confirming that the number of probed molecules in the contact area between the SThM tip and the sample are almost identical in each system. In addition, assuming that the elastic properties of the molecular films are similar,^8^ the contact mechanics and in turn the number of probed molecules in the contact area between the SThM tip and sample can be treated as similar regardless of the type of SAM system studied.

SThM measurements using contact and noncontact modes^8^ allowed us to evaluate the heat transport properties of the binary SAM domains. When a SThM tip is in physical contact with the SAM surface, a molecular junction is formed, giving rise to a heat transport channel. Consequently, heat in the tip dissipates through the channel, and the tip temperature decreases by Δ*T*. By measuring temperature with respect to tip displacement distance, *T*–Δ*z* curves that reflect heat transport properties of material are obtained (Figure S3). To facilitate the comparison between different molecules, we performed 2D mapping of Δ*T*^8^ on the patterned binary SAMs of **1**/**2**, **1**/**3**, and **1**/**4**. For 2D mapping, we avoided lateral probe scanning while in contact with the sample surface so as to minimize the potential influence of probe-surface contact mechanics on the thermal contrast. In addition, since the thermal contact resistance in SThM measurements can vary depending on the topography (i.e., film thickness), we focused on SAMs with nearly identical molecular lengths. The potential effect of adsorbed water on the SThM measurements is considered negligible due to the hydrophobic nature of the SAMs terminated with methyl or phenyl groups.^24^

The 2D Δ*T* map of binary **1**/**2** SAM displays a clear line-shape contrast (Figure 3a). As shown in Figure 3b, its cross-sectional profile (red curve) agrees well with the AFM topography image of the PDMS pattern (black curve) used for μ-CP to prepare the binary SAMs. This indicates that the Δ*T* contrasts observed for the 2D Δ*T* maps represent a temperature distribution of the domains. In the case of binary **1**/**2** SAM (Figure 3a), the domain of **1** is superior to that of **2** in terms of heat transport properties. Comparing to the line-shape pattern in the friction image (Figures 2b), the spatial resolution of the contact mode SThM imaging is lower, most likely due to the diffusive nature of heat. This reduction in spatial resolution is primarily due to the diffusive nature of heat at the nanometer scale, where heat spreads outside the SThM tip contact, resulting in a broader thermal profile. This phenomenon is particularly pronounced in contact mode due to the close thermal coupling between the tip and the sample. Unlike in the case of binary **1**/**2** SAM, binary **1**/**3** SAM scarcely displayed a domain-dependent image contrast in the 2D Δ*T* map (Figure 3c,d), indicating that the bond order (triple vs. double bonds) does not significantly affect the heat transport properties. On the other hand, the 2D Δ*T* mapping of binary **1**/**4** SAM (Figure 3e,f) showed increased heat transport properties for the domain consisting of **4** with an amide moiety compared to the domain of **1**. The Δ*T* contrast observed in Figure 3a–f is attributed to the heat transport properties of the SAM domains, which vary with the molecular backbone and bonding mode. Considering all the above results, the magnitude of the heat transport properties follows the order of **4** > **1** ≈ **3** > **2**.

**Figure 3:**
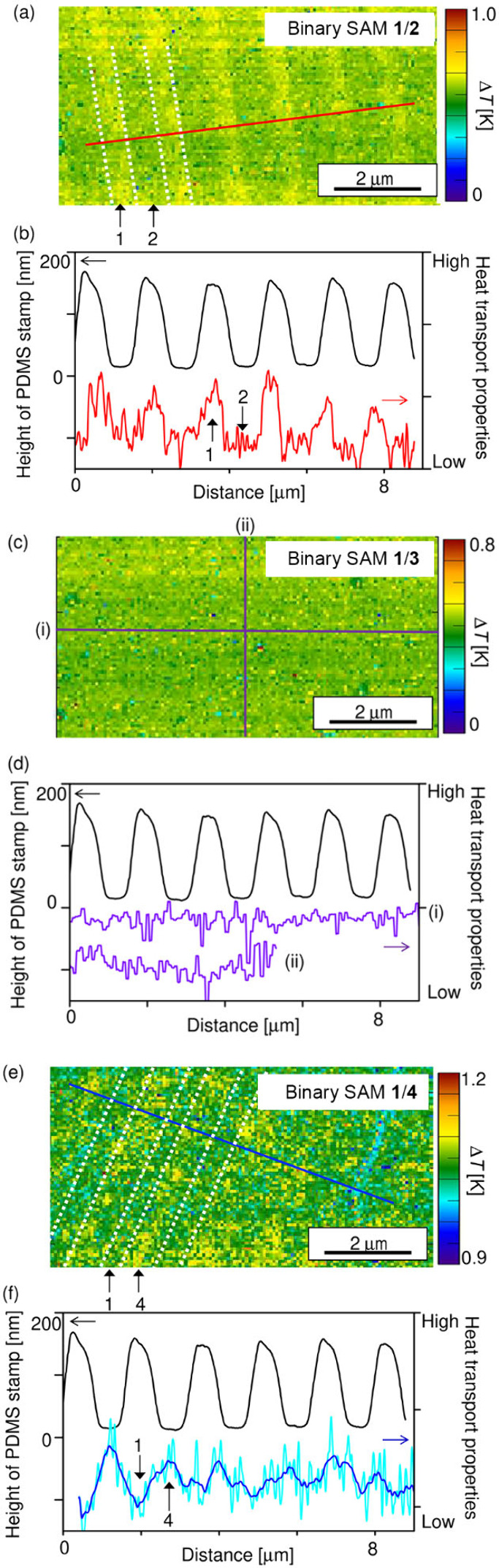
(a, c, e) 2D Δ*T* mapping for patterned binary SAMs of (a) **1**/**2**, (c) **1**/**3**, and (e) **1**/**4**. Dotted lines and arrows indicate line patterns and molecular species in the binary SAM domains. Raw data without guide lines are shown in Supporting Information (Figure S4). (b, d, f) Cross-sectional profiles of 2D Δ*T* maps for the binary (b) **1**/**2**, (d) **1**/**3**, and (f) **1**/**4** SAMs. The red and blue Δ*T* profiles in (b, f) are obtained from the solid lines in (a, e), respectively. The purple Δ*T* profiles (i) and (ii) in (d) are taken from the solid lines (i) and (ii) in (c), respectively. The Δ*T*-profile has been smoothed by a moving average in (f). In (b, d, e), black solid lines correspond to profiles for the topography of a PDMS pattern used for the μ-CP of the binary SAMs.

As reported previously,^8^ while contact mode SThM provides information about the heat transport properties of molecules, noncontact mode SThM can distinguish the thickness of binary SAM domains. While usual AFM requires contact between the probe and the sample surface, which is mechanically destructive, especially for soft organic materials, the noncontact method is completely mechanically nondestructive and allows high-resolution imaging of the temperature distribution associated with the thickness of each SAM domain.^8^ Although we also carried out noncontact SThM imaging for binary **1**/**2**, **1**/**3**, and **1**/**4** SAMs (Figure S5), as expected from the fact that the binary SAMs consist of molecules of nearly identical lengths, the SThM images obtained showed very weak contrast, if any.

Given the results of the 2D Δ*T* mapping, we can conclude that unsaturated C≡C and C=C bonds provide more effective pathways for heat transport than C–C bonds. It is interesting to note the subtle but substantial differences between **1** and **4** (Figure 3e,f). In compound **4**, the lone pair donation from the N atom to the carbonyl group gives rise to a partial π-bond character between the C and N atoms. Despite the similarity between **3** and **4** in terms of molecular backbone, there are differences in their heat transport properties. We attribute this to the presence or absence of intermolecular hydrogen bonding. In fact, it has been demonstrated that amide-containing thiols give well-ordered SAMs on Au(111), with formation of an extensive C=O···H–N hydrogen bonding network between amide moieties.^25,26^ This may be true for the SAM of **4**. When heat is dissipated from the tip to the Au surface, such hydrogen bonding is likely to provide intermolecular heat transport pathways,^27−31^ in addition to through covalent bonds, resulting in improved heat transport efficiency.

We previously reported that a *n*-butanethiol SAM exhibits better heat transport properties than a benzenethiol SAM, based on contact mode SThM measurements.^8^ However, in this study, the magnitude of the heat transport properties was evaluated to be **4** > **1** ≈ **3** > **2**. This discrepancy in the molecular backbone effect between the previous and present results obtained can be interpreted in terms of vibrational modes. The benzene group featuring a simple and rigid planar structure composed of fully delocalized double bonds has limited vibrational modes. When benzene rings are linked by chemical bonds, the number of vibrational modes available for heat transport increases. However, if molecular length becomes larger, it may itself generate thermal resistance.^8^ Therefore, to improve the heat transport properties of SAMs, it is important to strike a balance between the number of molecular vibrational modes involved in the heat transport pathways and the molecular length. The increased heat transport observed for certain domains in the present study is likely due to the presence of more efficient heat transport pathways with vibrational modes arising from π-bonds (**1** and **3**) and/or intermolecular hydrogen bonding (**4**), compared to those arising from σ-bonds alone (**2**).

In summary, SThM 2D temperature mapping of the binary SAMs of **1**–**4** (Figure 1) with nearly identical molecular lengths (i.e., SAM thickness) revealed an aspect of heat transport at the single molecule level, focusing on the chemical bonding mode. In terms of heat transport through the molecular backbone, multiple chemical bonds such as C≡C (**1**), C=C (**3**), and even a partial C–N double bond character (**4**) can facilitate more effectively heat transport compared to C–C bonds (**2**). Thus, the magnitude of the heat transport properties follows the order of **4** > **1** ≈ **3** > **2**. Importantly, the presence of intermolecular hydrogen bonding leads to an increase in the efficiency of heat transport. Since there are few examples of the experimental characterization of heat transport properties at the molecular level, the results obtained in this study may not only attract attention of theoreticians in the field of thermal properties, leading to an essential understanding of heat transport through organic molecules,^32,33^ but also promote the design of high-performance thermal interface materials (TIMs).

## Supporting Information Available

General, synthesis, preparation of binary SAM samples by μ-contact printing, scanning probe microscopy measurements, analytical data of new compounds (Figures S6–S9) (PDF)
