# Supplementary material for: Insights into Chemical Bonding Modes and Heat Transport at the Molecular Level
Source: J Phys Chem Lett. 2024 Oct 31;15(45):11189–93. doi: 10.1021/acs.jpclett.4c02325 (PMC11571208; doi:10.1021/acs.jpclett.4c02325)
Supplement: Supplementary file 1 — jz4c02325_si_001.pdf [file jz4c02325_si_001.pdf]

Supporting Information

Insights into Chemical Bonding Modes and Heat Transport at the Molecular Level

Shintaro Fujii,[†]* Yoshiaki Shoji,[‡,#] Yuma Masuda,[‡] Takanori Fukushima,[‡,#]*
and Tomoaki Nishino[†]*

[†]Department of Chemistry, School of Science, Tokyo Institute of Technology, 2-12-1 W4-10 Ookayama, Meguro-ku, Tokyo 152-8551, Japan
[‡]Laboratory for Chemistry and Life Science, Institute of Innovative Research, Tokyo Institute of Technology, 4259 Nagatsuta, Midori-ku, Yokohama 226-8501, Japan
[#]Research Center for Autonomous Systems Materialogy (ASMat), Institute of Innovative Research, Tokyo Institute of Technology, 4259 Nagatsuta, Midori-ku, Yokohama 226-8501, Japan

*To whom correspondence should be addressed.
E-mail: fujii.s.af@m.titech.ac.jp (S.F.); fukushima@res.titech.ac.jp (T.F.);
tnishino@chem.titech.ac.jp (T.N.)

1. General. Ethanol was purchased from Kanto Chemicals (Tokyo, Japan), and *n*-decanethiol (**2**) was purchased from Tokyo Chemical Industry (Tokyo, Japan). Compounds **1**,[S1] **3**,[S2] and 4-(tritylsulfanylmethyl)benzoic acid[S3] (**5**, Scheme S1) were synthesized according to the procedure reported previously. Au(111) substrates were prepared by thermal vacuum deposition of Au on mica substrates. Column chromatography was carried out using Silica Gel 60N (particle size: 63–210 μm). Infrared (IR) spectra were recorded at 25 °C on a JASCO model FT/IR-660*Plus* Fourier transform IR spectrometer. Nuclear magnetic resonance (NMR) spectroscopy measurements were carried out on a Bruker model AVANCE-400 spectrometer (^{1}H: 400.0 MHz and ^{13}C: 100.6 MHz). Chemical shifts (δ) are expressed relative to the resonance of the residual non-deuterated solvent for ^{1}H ($CDCl_3$: ^{1}H(δ) = 7.26 ppm) and the resonance of the residual solvent for ^{13}C ($CDCl_3$: ^{13}C(δ) = 77.16 ppm). Absolute values of the coupling constants are given in Hertz (Hz), regardless of their sign. Multiplicities are abbreviated as singlet (s), doublet (d), triplet (t), quartet (q), multiplet (m) and broad (br). Mass spectrometry measurements were carried out on a Bruker model micrOTOF II mass spectrometer equipped with an atmospheric pressure chemical ionization (APCI) probe.

2. Synthesis.

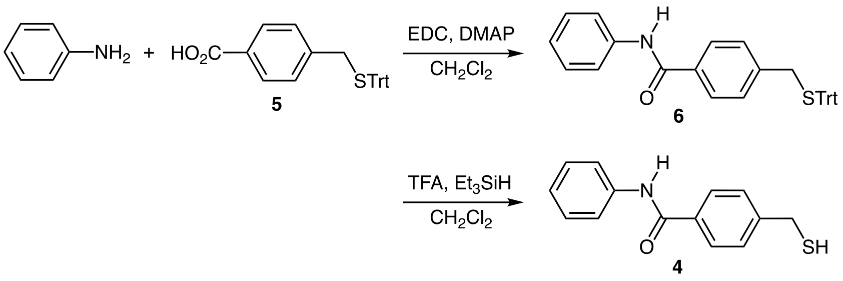

Scheme S1. Synthesis of compound **4**. Trt = triphenylmethyl.

Compound 6. Under a nitrogen atmosphere at 25 °C, aniline (75 mg, 0.81 mmol) was added to a CH_2Cl_2 solution (8.0 mL) of a mixture of 4-(tritylsulfanylmethyl)benzoic acid **5**[S3] (500 mg, 1.2 mmol), 4-dimethylaminopyridine (DMAP) (316 mg, 2.6 mmol), and 1-(3-dimethylaminopropyl)-3-ethylcarbodiimide (EDC) (214 mg, 1.4 mmol), and the resulting mixture was stirred at 25 °C for 16 h. The reaction mixture was poured into water and extracted with CH_2Cl_2. The combined organic extract was washed with water and brine, dried over anhydrous $MgSO_4$, and evaporated to dryness under reduced pressure. The residue was subjected to column chromatography on SiO_2 (*n*-hexane/ethyl acetate 7/1 v/v) to allow isolation of **6** (202 mg, 0.42 mmol) as a white powder in 52 % yield. FT-IR (KBr): ν (cm^{-1}) 3393, 3080, 3054, 3032, 3016, 2957, 2925, 2852, 1658, 1597, 1524, 1505, 1486, 1439, 1413, 1394, 1316, 1252, 1200, 1187, 1156, 1098, 1077, 1034, 1019, 1001, 980, 964, 933, 906, 884, 862, 837, 801, 769, 753, 742, 702, 689, 674, 643, 616. ^{1}H NMR (400 MHz, acetone-d_6, 25 °C): δ (ppm) 9.47 (s, br, 1H), 7.90 (d, J = 8.1 Hz, 2H), 7.84 (d, J = 8.1 Hz, 2H), 7.50 (d, J = 7.9 Hz, 6H), 7.41–7.28 (m, 13H), 7.11 (t, J = 7.5 Hz, 1H), 3.44 (s, 2H). ^{13}C NMR (100 MHz,

$CDCl_3$, 25 °C): δ (ppm) 166.1, 145.8, 142.0, 140.5, 135.2, 130.6, 130.1, 129.6, 129.0, 128.7, 127.9, 124.7, 121.1, 68.5, 37.3. APCI-TOF mass: calcd. for $C_{33}H_{27}NOS$ $[M+H]^+$: m/z = 486.1886; found: 486.1931. ^1H and ^{13}C NMR spectra of **6** are shown in Figures S6 and S7, respectively.

Compound 4. Under a nitrogen atmosphere at 25 °C, trifluoroacetic acid (TFA) (200 μL, 2.6 mmol) was added to a CH_2Cl_2 solution (5.0 mL) of a mixture of compound 6 (100 mg, 0.21 mmol) and Et_3SiH (34 μL, 0.21 mmol), and the resulting mixture was stirred at 25 °C for 1 h. The reaction mixture was poured into water and extracted with ethyl acetate. The combined organic extract was washed with water and brine, dried over anhydrous $MgSO_4$, and evaporated to dryness under reduced pressure. The residue was subjected to column chromatography on SiO_2 (n-hexane/CH_2Cl_2 1/2 v/v) to allow isolation of 4 (44 mg, 0.18 mmol) as a white powder in 86 % yield. FT-IR (KBr): ν (cm^{-1}) 3353, 3059, 2957, 2924, 2852, 2537, 1656, 1599, 1573, 1529, 1508. 1439, 1413, 1324, 1303, 1261, 1178, 1107, 1073, 1019. 976, 908, 887, 854, 749, 712, 688, 644, 626. ^1H NMR (400 MHz, acetone-d_6, 25 °C): δ (ppm) 9.49 (s, br, 1H), 7.96 (d, J = 8.1 Hz, 2H), 7.86 (d, J = 8.1 Hz, 2H), 7.51 (d, J = 8.1 Hz, 2H), 7.37 (dd, J = 7.2, 8.1 Hz, 2H), 7.12 (t, J = 7.2 Hz, 1H), 3.86 (d, J = 8.0 Hz, 2H), 2.31 (t, J = 8.0 Hz, 1H). ^{13}C NMR (100 MHz, acetone-d_6, 25 °C): δ (ppm) 166.0, 146.4, 140.3, 134.80, 129.5, 129.0, 128.6, 124.5, 121.0, 28.6. APCI-TOF mass: calcd. for $C_{14}H_{13}NOS$ $[M+H]^+$: m/z = 244.0791; found: 244.0712. ^1H and ^{13}C NMR spectra of **4** are shown in Figures S8 and S9, respectively.

3. Preparation of Binary SAM Samples by μ-Contact Printing. A commercial recordable compact disk (CD-R), which had a series of stripe-like convexities with a periodic pattern of approximately 1~2 μm, was used as a mold to prepare the stamp for μ-contact printing (μ-CP). The mold pattern was transferred to a poly(dimethylsiloxane) (PDMS; Dow-Corning SYLGARD silicon elastomer 184) stamp. μ-CP with a wet-inking method was carried out by applying several drops of an ethanolic solution of **1** (~1 mM) to the PDMS stamp surface; after 30 sec, excess solution was removed from the inked stamp. The inked stamp was placed on an Au(111) substrate and allowed to stand for 30 sec. When the PDMS stamp was removed from the substrate, a molecular layer of **1** was left on the areas of Au(111) that had been in contact with the stamp. The Au(111) surface patterned with **1** was then immersed in an ethanolic solution of **2** (1 mM) for 10 min. The resulting Au(111) substrate was rinsed with pure ethanol and dried in air, giving a binary SAM sample of **1**/**2** (Figure S1). Patterned binary SAM samples of **1**/**3** and **1**/**4** were also prepared by similar methods.

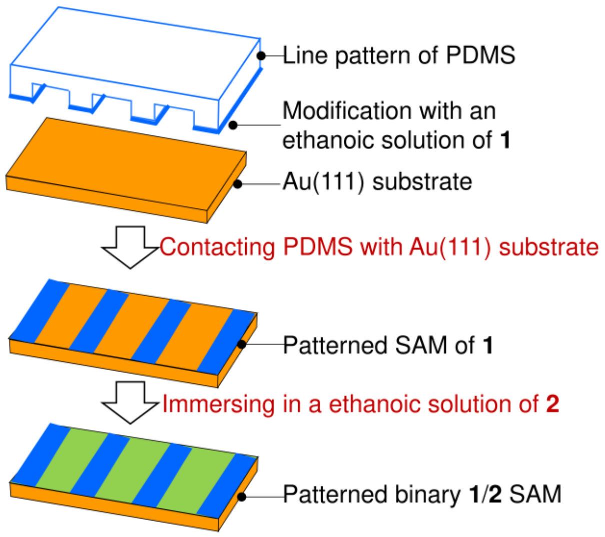

Figure S1. Schematic illustration of the μ-contact printing method. A linear pattern of one thiol (**1**) molecule was transferred to Au(111) using a polydimethylsiloxane (PDMS) stamp, and the modified Au(111) substrate was then immersed in an ethanoic solution of another thiol molecule (**2**), resulting in a linear pattern of SAM(**1**) and SAM(**2**).

4. Scanning Probe Microscopy Measurements. SThM measurements were carried out using a Nanoscope V controller (Bruker, Santa Barbara, CA, United States) and VertiSense SThM imaging amplifier with a VTP-200 cantilever tip (Applied Nanostructures, Mountain View, CA, United States) under air at ambient temperature. SThM cantilever tips (nominal spring constant of 10 N/m) having a thermocouple junction at the apex were used for two-dimensional (2D) mapping of force–distance and relative temperature–distance curves. In the SThM measurements, the alignment of a laser (690 nm, 1 mW) irradiated on a tip was adjusted to heat the tip to approximately 80~99 °C. SThM measurements in contact and noncontact modes were performed according to previously established procedures.[S4] AFM topographic and friction images in contact mode were measured using a Nanoscope V controller and VTP-200 cantilever tip. STM topography was recorded under constant-current mode on a Nanoscope 3D controller (Bruker, Santa Barbara, CA, United States) using mechanically cut Au tips. All the AFM and STM topography data were recorded under air.

5. Supporting Figures S2–S9.

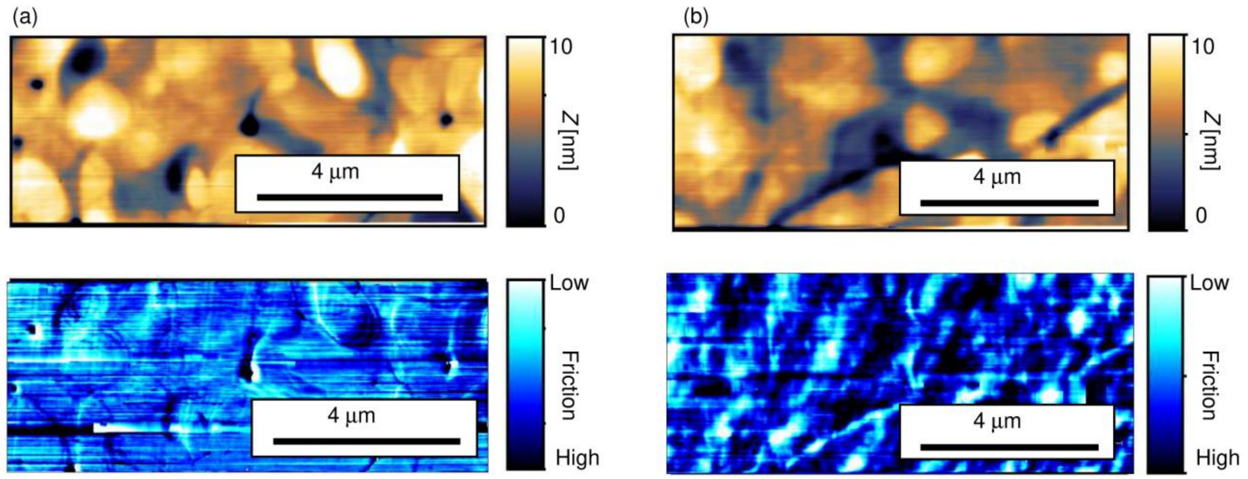

Figure S2. AFM topographic and friction images of patterned binary (a) **1**/**3** and (b) **1**/**4** SAMs.

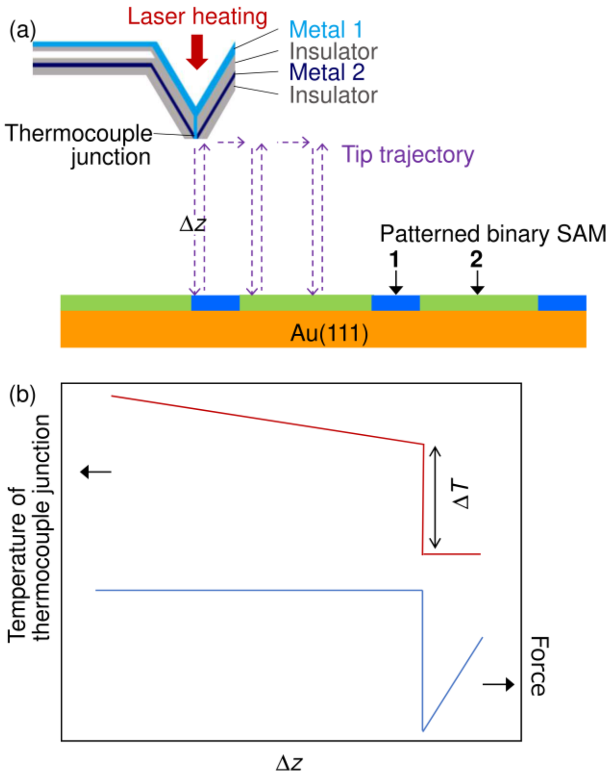

Figure S3. (a) Schematic illustration of the setup of SThM measurements for a binary SAM, where the tip trajectory is indicated by arrows. (b) Schematic diagram of F−Δz and T−Δz curves. When physical contact between a heated tip and a surface occurs, the temperature suddenly drops, while the force drops once due to attractive van der Waals forces then increases due to repulsive forces. After contact, a heat transport channel is formed from the tip to the surface. Consequently, heat in the tip dissipates though the heat transport channel, and the temperature of the tip decreases by ΔT. By measuring ΔT in a T−Δz curve, information on heat dissipation through a SAM, which is related to the heat transport properties of SAMs, can be obtained.[S4]

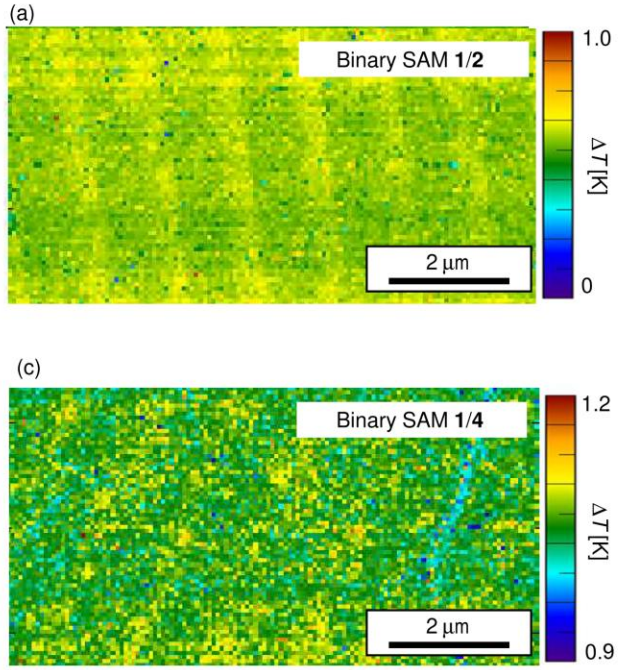

Figure S4. 2D ΔT mapping for patterned binary SAMs of (a) **1**/**2**, (b) **1**/**3**, and (c) **1**/**4** using contact mode.

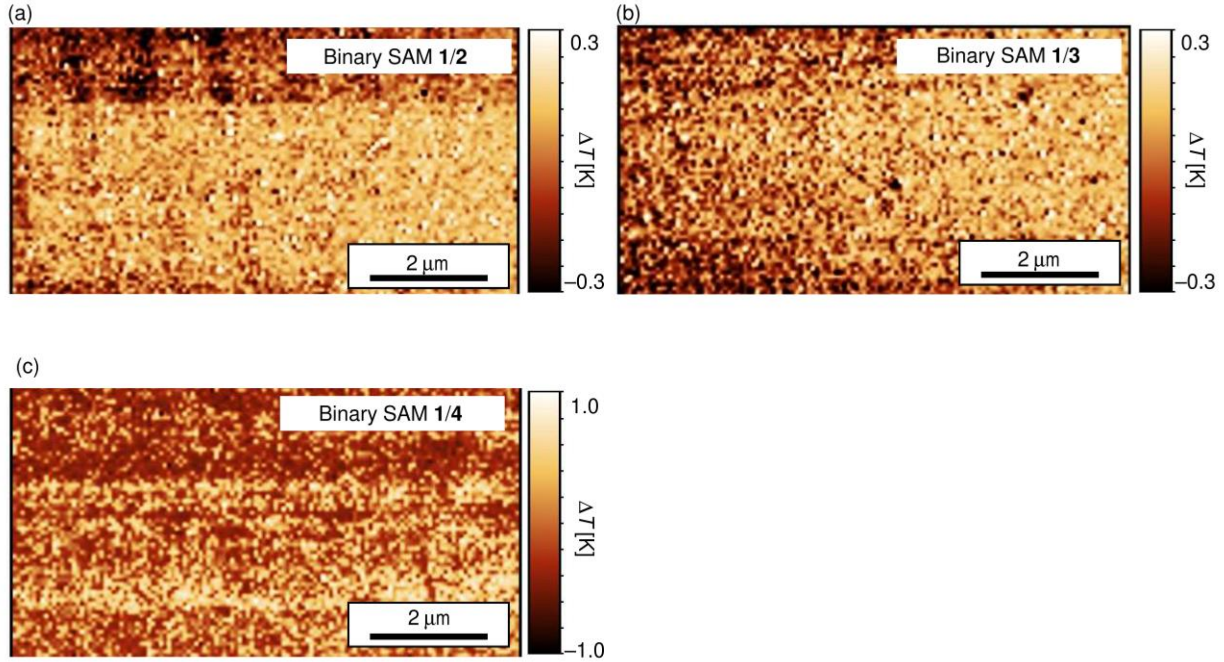

Figure S5. 2D ΔT mapping for patterned binary SAMs of (a) **1**/**2**, (b) **1**/**3**, and (c) **1**/**4** using noncontact mode.[S4]

6. Analytical Data of New Compounds.

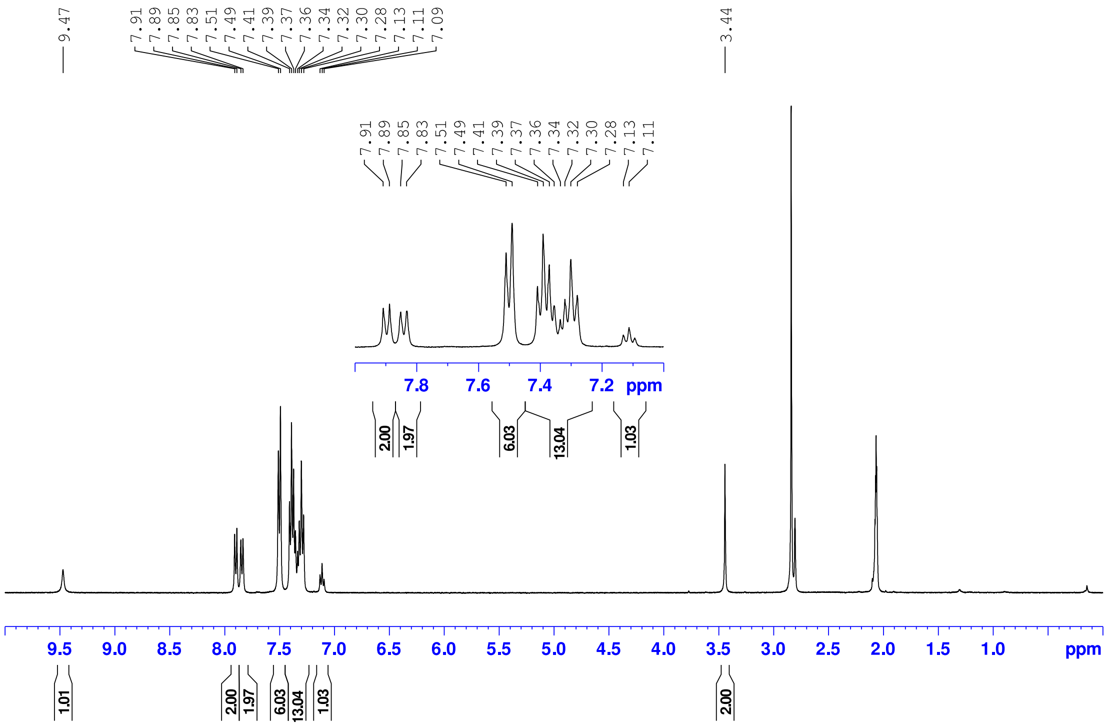

Figure S6. ^{1}H NMR spectrum (400 MHz) of **6** in acetone-d_6 at 25 °C.

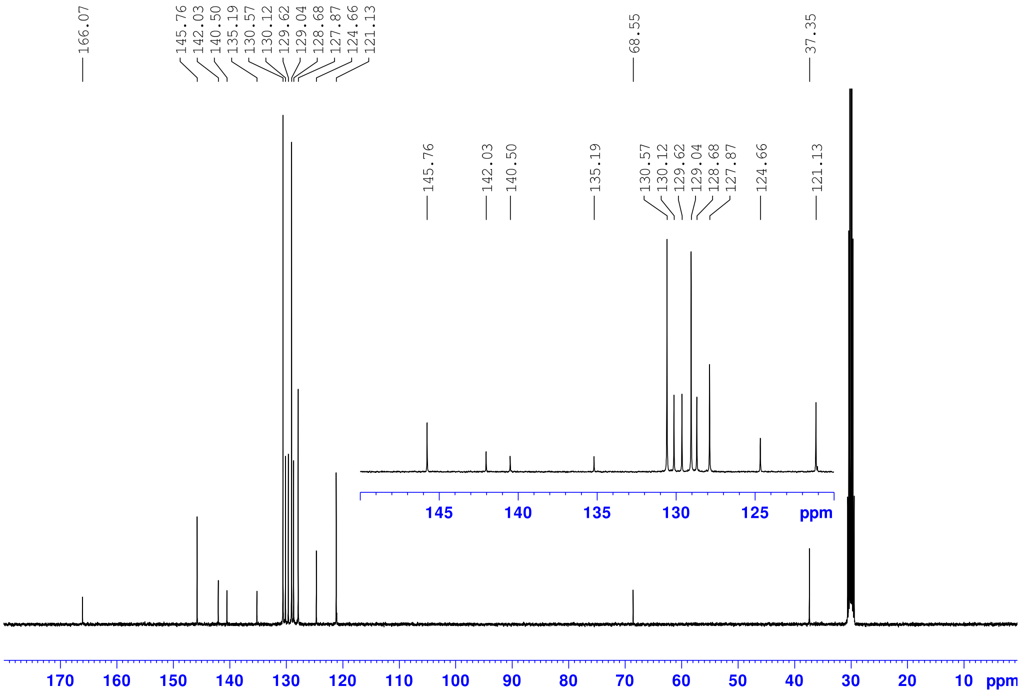

Figure S7. ^{13}C NMR spectrum (100 MHz) of **6** in acetone-d_6 at 25 °C.

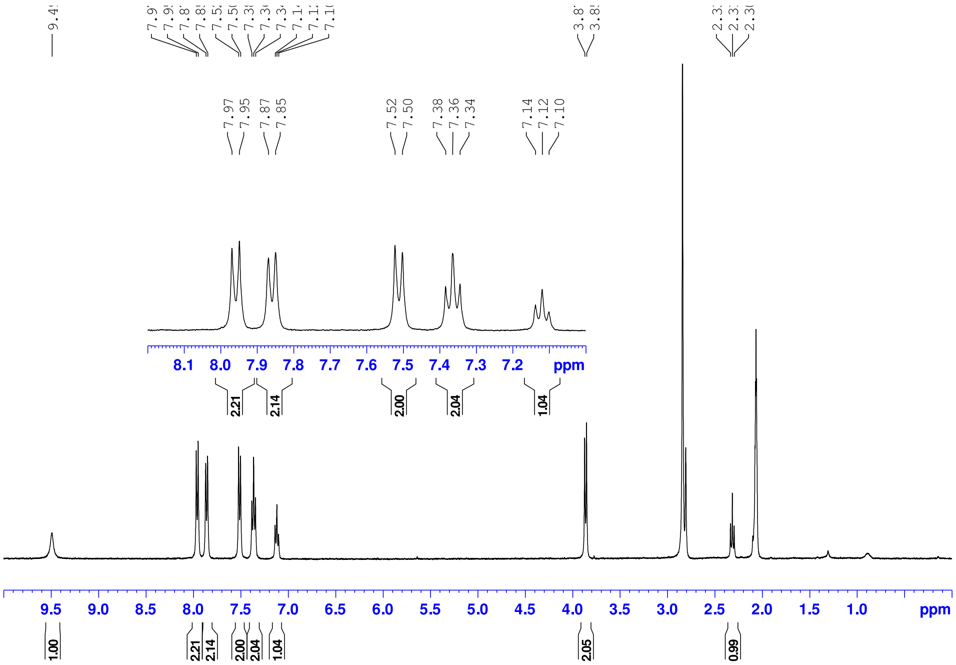

Figure S8. ^{1}H NMR spectrum (400 MHz) of **4** in acetone-d_6 at 25 °C.

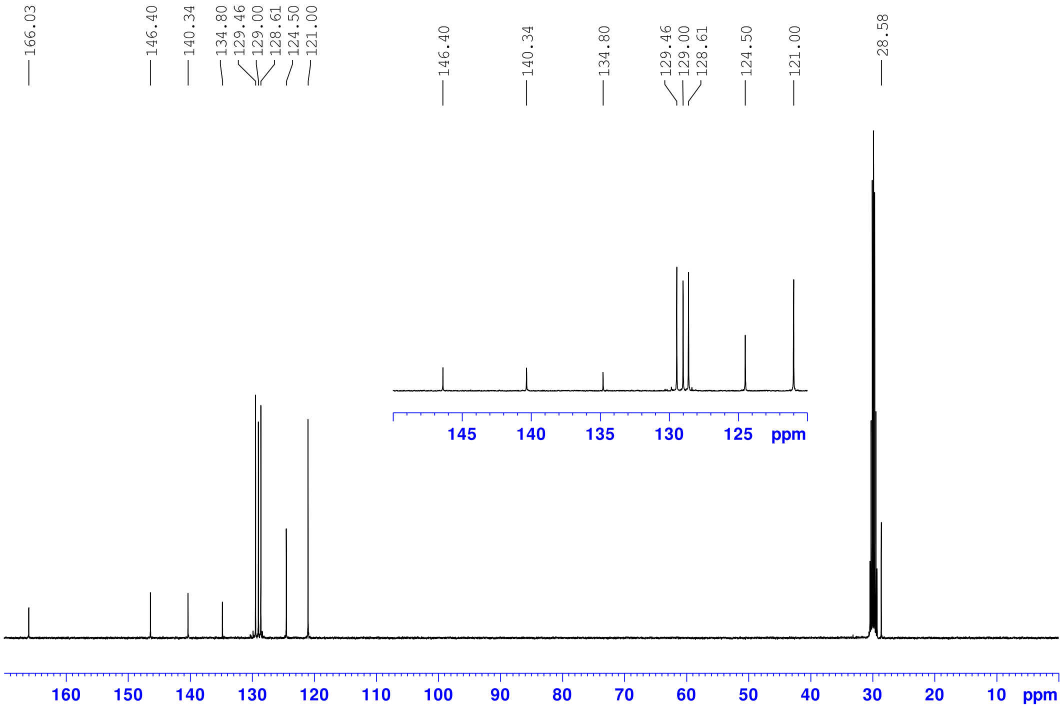

Figure S9. ^{13}C NMR spectrum (100 MHz) of **4** in acetone-d_6 at 25 °C.

7. Supporting References.

S1. Acharya, A.; Mete, T. B.; Kumari, N.; Yoon, Y.; Jeong, H.; Jang, T.; Song, B.; Choi, H. C.; Han, J. W.; Pang, Y.; Yun, Y.; Kumar, A.; Lee, I. S. Ultrathin Covalent Organic Overlayers on Metal Nanocrystals for Highly Selective Plasmonic Photocatalysis. *Nat. Commun.* **2023**, *14*, 7667.

S2. Xiao, T.; Ye, Q.; Sun, L. Hunting for the Active Sites of Surface-Enhanced Raman Scattering: A New Strategy Based on Single Silver Particles. *J. Phys. Chem. B* **1997**, *101*, 632–638.

S3. Bugaut, A.; Jantos, K.; Wietor, J.-L.; Rodriguez, R.; Sanders, J. K. M.; Balasubramanian, S. Exploring the Differential Recognition of DNA G-Quadruplex Targets by Small Molecules Using Dynamic Combinatorial Chemistry. *Angew. Chem. Int. Ed.* **2008**, *47*, 2677–2680.

S4. Fujii, S.; Shoji, Y.; Fukushima, T.; Nishino, T. Visualization of Thermal Transport Properties of Self-Assembled Monolayers on Au(111) by Contact and Noncontact Scanning Thermal Microscopy. *J. Am. Chem. Soc.* **2021**, *143*, 18777–18783.
